# Medical Fitness in workers suffering from mixed anxiety-depressive disorders:

**DOI:** 10.1192/j.eurpsy.2023.1852

**Published:** 2023-07-19

**Authors:** L. Ben Afia, G. Bahri, H. Ben said, H. ziedi, M. Mersni, D. Brahim, S. Ernez, N. Mechergui, I. Youssef, N. Ladhari

**Affiliations:** Occupational medecine department, Charles Nicolle Hospital, Tunis, Tunisia

## Abstract

**Introduction:**

Anxiety and depressive disorders are major public health problems associated with multiple adverse occupational outcomes, including unemployment, reduced productivity, and absenteeism.

**Objectives:**

To study the socio-professional and medical characteristics of workers with mixed anxiety -depressive disorders and to evaluate their impact on work ability.

**Methods:**

A descriptive and retrospective study conducted in the occupational medicine department at Charles Nicolle Hospital, involving all the medical records of workers suffering from mixed anxiety –depressive disorders that were referred for a medical opinion of fitness for work from January 1, 2014, to December 31, 2020.

**Results:**

The study included 62 females and 20 males diagnosed with mixed anxiety-depressive disorders with a mean age: 41.4± 8 years. The average professional seniority was 12.8 years±7.8 years. The most auspicious occupational sectors for these disorders were health (41%) and communication (30%). Most of these workers (62%) were fit for work with professional restrictions (10 workers to positions with a lower mental load and 20 exclusions from night shift work), though 12% were declared unfit for work temporarily. Twenty-one workers were fit to continue working and one worker was unfitted to work.

The overall prevalence of mixed anxiety –depressive disorders was found to be significantly elevated in female patients (p: <0.001).

**Conclusions:**

The decision of medical fitness for work among workers with psychiatric disorders considers their physical and mental capacities as well as the conditions in which the work is carried out, aiming to annihilate the risk of psychic imbalance. Thus, an adjustment of workstations can be an important determinant in the prevention of psychosocial risks.

**Disclosure of Interest:**

None Declared

